# Mr. Creaser, on Vaccination

**Published:** 1806-04

**Authors:** Thomas Creaser

**Affiliations:** One of the Surgeons of the Royal Somerset Jennerian Society


### *To the Editors of the Medical and Physical Journal.*

GENTLEMEN,

I HAVE transmitted to you as above, the Report of the proceedings of the Royal Somerset Jennerian Society during the past year; very ardently I hope, that a success in the train of example of the county of Sussex may lead to similar measures of every county in the united kingdom. The appointment of the Duke of Bedford (whose munificent patronage of all the arts which contribute to the welfare of mankind, and whose zeal in favour of vaccination as President of the Royal Jennerian Society is so honorably bly conspicuous) to the government of Ireland, will no doubt give sanction and extension to this invaluable practice in the sister kingdom.

The results of our practice would have been communicated to you without a comment, but that I am led to make some in consequence of perusing the Report of Dr. Wood, of Newcastle, on the progress and success of vaccination in that town, in your Journal for February last.

The example of some controversies in your Journal, would certainly lead me to the careful avoiding of similar engagements, on any question which did not involve the interests of important and philosophical truth; such I consider to be concerned in the opinions which Dr. Wood has expressed, in opposition to Dr. Jenner's most ingenious and sagacious observations on the influence of herpetic eruptions of the skin, in altering the character and appearance of the vaccine pustule. Dr. Wood's ideas on this point are thus expressed. "I can now also, with some confidence, advance my opinion, though contrary to some late prevailing opinions, that some other eruptions, herpes for instance, do not prevent or lessen this security. I have been cautious in advancing this opinion, though founded on my experience and frequent observation, because Dr. Jenner has advanced a different opinion." I have on the contrary, been accused by a Mr. Clement, in No. 75 of your Journal, of not producing in my former report, "those sterling instructions as well as observations on this particular point that Dr. Jenner had given. I apprehend that those instructions are only sterling, which are the result of much and accurate observation; or that those observations only are of value, that are derived from experience."

This latter sentence of Dr. Wood contains a truism in which I most heartily and perfectly concur, and it is on this basis that I join issue with him. How far he may have been more cautious in the demonstration of his opinions, because they militated against those of Dr. Jenner, I confess that, from the spirit of his remarks, I am not a little sceptical.

It is not my intention, Gentlemen, to enter into any extended pathological discussion on the subject, but to refer to the "result of much and accurate observation," and the evidence of facts.

Besides the 354 cases of perfect vaccination, reported by the Royal Somerset Jennerian Society in Bath, there occurred occurred eleven cases of spurious pustule. Of these, *nine actually took place in subjects considerably affected with herpes, and as such, were recorded at the time in our register.* Two more of the eleven persons had been insusceptible of small-pox, but had also spurious pustules. Two more were *perfectly insusceptible* of the vaccine disorder, who were considerably affected with herpes. Our Register is directed equally by myself and my colleague, Mr. Tudor, and, as well as the inoculated persons, is open to the inspection of superintending physicians and gentlemen, members of the Society.

As the term, *spurious pustule,* has been a subject of much philological quibbling, I will give it such a definition as may be grappled with. It is the most ordinary and uniform deviation from the appearance and action of the vaccine pustule; it has a premature, more diffused, and differently coloured inflammation; at an early period it contains purulent matter, and desiccates into a soft crustaceous scab. Such appearances I affirm to constitute the *ordinary variety* of effect produced by the inoculation of vaccine virus; and such I contend, in the most accurate language of pathology, may be designated as *spurious pustules.* As far as the instances above quoted tend, I believe their causation by herpes to be certain, and I could prove the same by much greater personal experience. The question is too recent to admit of reference to authorities, but I will adduce an extract from the proceedings of the Institution, to promote Cow-Pox Inoculation in Nottingham, by Dr. Clark, in your Journal for March ult.

"We have in about ten cases, observed a peculiar scab following a regular vesicle. It is *formed earlier* than the usual scab, but I am quite at a loss for words to describe it so as to be understood. It is of a greyish brown colour, laminated; considerably elevated above the surface of the cuticle, and about four times the dimensions of the common scab; its edges are ragged, and its surface rough; *the persons on whom it is observed, have been most commonly scorbutic.*" Dr. Clarke here, no doubt, employs the term scorbutic in its more usual acceptation, that is, to denote cuticular disease; and in this meaning, his description is much in proof of my argument on the question.

At a period of active hostility, between the vaccinists and their unprincipled opponents, I most sincerely deprecate the existence of a difference of sentiment, which is marked with the character of party, and which I ardently wish to see converted into unanimity of exertion, against the the common enemy. I allude to the panegyrics of Dr. Wood on Dr. Pearson's services in the cause of the Vaccina. To the "Statement of Evidence by the Vaccine Pock Institution," I with much pleasure admit the interests of vaccination to be essentially indebted. On the history of some early proceedings in vaccination, I have already given my sentiments,* and it would be as repugnant to my present, as my former conviction, to retract them. If these should, by some, be deemed partial, weak, or incorrect, I am proud of perceiving a coincidence of opinion, in the able, spirited, and argumentative "Reply to Anti-Vaccinists," by Mr. Moore.

I am, &c.

THOMAS CREASER,

One of the Surgeons of the Royal Somerset Jennerian Society.

---

### *Royal Somerset Jennerian Society.*

* Vide Observations on Dr. Pearson's Examination of the Report of the Vaccine Pock Committee of the House of Commons, second Edition. —Murray.

